# Editorial: Translating artificial intelligence into clinical use within cardiology

**DOI:** 10.3389/fcvm.2022.995234

**Published:** 2022-08-04

**Authors:** Paul Leeson, Shane Nanayakkara, Pablo Lamata

**Affiliations:** ^1^Cardiovascular Clinical Research Facility, RDM Division of Cardiovascular Medicine, University of Oxford, Oxford, United Kingdom; ^2^Department of Cardiology, The Alfred, Melbourne, VIC, Australia; ^3^Baker Heart and Diabetes Institute, Melbourne, VIC, Australia; ^4^Department of Biomedical Engineering, School of Biomedical Engineering and Imaging Sciences, King's College London, London, United Kingdom

**Keywords:** artificial intelligence, cardiology, heart failure, cardiovascular imaging, disease prediction

## Introduction

In the foreword to “The Seeds of Artificial Intelligence,” Gregory Freiherr sets out a vision for artificial intelligence in medicine (1): “*..the intelligent machine, a device that mimics the expert's reasoning power and can retain in retrievable power much of the knowledge currently available to experts in a given specialty*.” He also set out a timetable for when these systems could reach clinical practice: “*Most systems of this type are still immature. But some are already moving into the real world and others will make the transition within the next few years*.” The book was written in 1980 and the “*next few years*” has extended to “*40 years*” but we have now, arguably, reached that threshold, where artificial intelligence is at a stage of development when it could transition into routine application in medicine (2).

Throughout this period cardiology, and in particular cardiovascular imaging, has been at the forefront of understanding how to apply computational approaches to common medical tools such as an ECG recording or an ultrasound image (3). Now there is a final hurdle to overcome to ensure these solutions impact clinical practice. How do we translate them effectively into regular clinical use? This Research Topic was designed to provide an opportunity for authors to present their solutions to this problem. The topic was kept broad to attract submissions about both innovations and applications and, across the wide variety of papers submitted, several themes have emerged. These reflect the most active academic areas in the application of artificial intelligence to cardiovascular medicine.

## Cardiovascular imaging and cardiovascular magnetic resonance

Cardiovascular imaging has been an exemplar area of cardiovascular research for adoption of artificial intelligence (3). The volume of data and natural collaborations between academic centers internationally have allowed testing and development of different approaches using large scale patient datasets. Research efforts such as UK Biobank, with hundreds of thousands of freely available cardiovascular magnetic resonance image datasets have also accelerated development of solutions (4). The analysis of images using artificial intelligence is also central to many of the secular applications of artificial intelligence during for example internet searches. Therefore, the technology solutions are well-advanced. In this Research Topic, Sanchez-Martinez et al. provide a review that brings together understanding of how machine learning can be applied to decision making in cardiac imaging. Furthermore, four new technical approaches to segment and analyse cardiovascular magnetic resonance scans for different disease states are presented. Approaching the problem from a more generalized angle, Vergani et al. present a deep learning approach for classification of images while Huellebrand et al. provide a holistic analysis of methods for disease classification. More focused on specific cardiovascular magnetic resonance sequences, Sharma et al. present a method for classification of myocardial scar identified with late gadolinium imaging, a stalwart of the modality for the last 20 years. In comparison Gonzales et al. present a new way to manage automation of T1 mapping, an emerging option for gadolinium-free scar imaging. Together these manuscripts describe innovations across the whole pipeline of image handling from image sorting and classification to disease identification.

## Signal processing in cardiovascular medicine

Technological solutions using artificial intelligence, however, clearly extend beyond image handling. Intelligent signal processing is an area of rapid development and this has been effectively presented in several papers within the Research Topic. Electrophysiology is well-known for its analysis of ECG signals and Herrero Martin et al. present a framework for characterization of signals to define cardiac disease. Three others paper approach another well-known signal processing problem, the characterization of cardiac and vascular pressures. Yamanaka et al. have tackled a problem with a very large potential application footprint. Blood pressure measurement is relevant to both the assessment of health and for the identification or monitoring of disease. The ability to do this without the need of an inflatable cuff would transform daily practice and their early data shows new ways for cuffless blood pressure estimation. Westphal et al. take a different approach to understanding pressures within the cardiovascular system, with a similarly widespread potential clinical footprint. Heart sounds are readily accessible with a stethoscope but the ability to estimate left ventricular pressure based off heart sounds is a novel application of machine learning. The other region of the cardiovascular system in which pressures and stresses can have important clinical implications is the aorta. Increased aortic shear stress has been implicated in aortic remodeling as well as dissection and atheroma development. Ferdian et al. present new data on technological approaches to estimate aortic shear stress from simply acquired parameters. These solutions provide new approaches to extract information from clinical signals. Several of these measures are not in routine clinical use but if these methods increase availability they may encourage translation into practice.

## Heart failure diagnosis and management

With regard to disease areas, heart failure has proved to be the most popular for application of new artificial intelligence management approaches within our Research Topic. Heart failure represents the end stage of most cardiac conditions and has a heterogenous pathology with numerous potential management approaches. Identifying the type of heart failure is critical and identifying the disease process early is important to prevent irreparable damage. Identification of heart failure has been addressed in three papers in this Research Topic. Two focus on imaging with Fletcher et al. having reviewed the possibility for artificial intelligence to augment the power of echocardiography for identification of heart failure with preserved ejection fraction, while, Asher et al. present a review covering the possibility for imaging classification of dilated cardiomyopathy phenotypes. In contrast, Alkhodari et al. have explored whether deep learning based on patient records is effective for classification of heart failure profiles. Finally, Kenig et al. have tackled the question of management approaches and present an algorithm for assessment and titration of diuretic treatment in heart failure. Heart failure is an increasing problem within healthcare and the reviews suggest there remains a huge untapped potential for applications of artificial intelligence in this space.

## Disease characterization, prognosis, and prediction

In a final theme of the Research Topic four papers address the ongoing question of how artificial intelligence can be applied to characterize disease and predict outcomes. These papers cover an extensive range of topics and provide some of the most challenging potential applications. The ability to predict the future and identify patients at high risk is hugely difficult but may be tractable with artificial intelligence processing techniques. Zhou et al. have simply modeled using existing data what happens to patients who have pulmonary hypertension to try and understand who may be most at risk of frailty-related problems. Wang et al. have also used patient-related data to look at outcomes from Coronary Care Units and Jiang et al. have adopted the same approach to look at outcomes after mitral valve surgery. These papers show promise for predictive algorithms based on patient acquired data but further clinical validation is required in independent datasets.

## Conclusion

When we proposed this Research Topic we had a vision to present a broad range of papers promoting real world clinical applications of artificial intelligence in medicine. We were unsure of what would emerge from the clinical and academic community but the spread of topics and technical solutions is impressive. Interestingly, many are not yet ready for clinical application and there is clearly still some way to go to achieve our goal of clinically translatable artificial intelligence in cardiology. However, the first steps are being made and we look forward to a time when, in Eric Topol's words (2): “*Eventually, doctors will adopt AI and algorithms as their work partners. This leveling of the medical knowledge landscape will ultimately lead to a new premium: to find and train doctors who have the highest level of emotional intelligence*.”

## Author contributions

PLe drafted the manuscript. SN and PLa edited for important intellectual content. All authors contributed to the article and approved the submitted version.

## Funding

PLe was supported by the Oxford NIHR Biomedical Research Centre and Oxford BHF Centre for Research Excellence.

## Conflict of interest

PLe is a founder and shareholder of Ultromics, an AI imaging company, and has patents related to clinical applications of AI. The remaining authors declare that the research was conducted in the absence of any commercial or financial relationships that could be construed as a potential conflict of interest.

## Publisher's note

All claims expressed in this article are solely those of the authors and do not necessarily represent those of their affiliated organizations, or those of the publisher, the editors and the reviewers. Any product that may be evaluated in this article, or claim that may be made by its manufacturer, is not guaranteed or endorsed by the publisher.
